# Revisiting PINI Scoring in Light of Recent Biological Advances

**DOI:** 10.3390/nu15081846

**Published:** 2023-04-12

**Authors:** Yves Ingenbleek

**Affiliations:** Laboratory of Nutrition, Faculty of Pharmacy, University of Strasbourg, Route du Rhin, 67401 Illkirch-Graffenstaden, France; ingen@unistra.fr; Tel.: +33-467748717

**Keywords:** WHO, developing countries, malnutrition, inflammation, biomarkers, lean body mass, transthyretin, retinol, iron

## Abstract

The prognostic inflammatory and nutritional index (PINI) is a simple scoring formula allowing the follow-up of dietary protein restriction and infectious complications affecting critically ill patients hospitalized in medical and surgical wards. The World Health organization (WHO) has recently recommended using the binary CRP (C-reactive protein) and AGP (α_1_-acid glycoprotein) numerators of the PINI formula in underprivileged inhabitants of developing countries to evaluate their (sub)clinical infectious states making their chronic malnutrition worse. These studies, mainly located in Africa and Asia, demonstrate that children and women enduring the combined effects of infectious burden and (micro)nutrient deprivation (principally retinol and iron) usually manifest persistent refractoriness and slackened recovery throughout dietary rehabilitation. The additive measurement of ALB (albumin) and TTR (transthyretin) composing the denominator of the PINI formula is shown to be helpful in grading the downsizing of lean body mass (LBM), a cornerstone of bodybuilding. The confrontation of these four objective parameters thus allows the quantification of the respective importance of nutritional and inflammatory components of any disease process, taking into account that TTR is the sole plasma protein remaining highly correlated to the fluctuations of LBM. The below review highlights the prevailing roles played by protein nutritional states in the release of plasma retinol to target tissues and to the restoration of iron-deficient anemias.

## 1. Introduction

The proposal to provide a simple and useful PINI formula grading the level of both dietary and infectious conditions affecting critically ill hospitalized patients was made in 1985 at the University of Dakar, Senegal (West Africa). The formula is a ratio employing the blood indices currently used for the assessment of nutritional states as the denomincator (albumin [ALB] and transthyretin [TTR]) and those biomarkers (C-reactive protein [CRP] and α_1_-acid glycoprotein [AGP]) prevailing in the detection and scaling of inflammatory processes as the numerator. The selection of CRP and AGP for the scoring formula was achieved after stepwise discriminant and canonical comparison with five other different acute-phase proteins (APPs), namely haptoglobin, ceruloplasmin, fibrinogen, α_1_-antitrypsin, and α_1_-antichymotrypsin. This screening approach was completed by expert statistical interpretation of the recorded data [1].

In control subjects, PINI values are close to 0 but rise progressively in ambulatory subjects suffering minor stresses, further increasing in severely diseased patients in proportion to their nutritional deficits and/or superimposed inflammatory burden. The numeric encoding of individual PINI values paves the way allowing the calculation of a Global Health Score (GHS) hereby stratified (see Appendix A). During the last decades, the PINI formula was successfully utilized in nearly 400 studies. Indeed, it is exemplified by a dozen investigations performed on sick patients regardless of age, gender, and disease states: children enduring varying morbid processes [2], burns [3], or surgery [4]; adult subjects undergoing trauma management [5], lung [6], and kidney [7] rehabilitation processes in intensive care units [8]; adults suffering from several types of cancer [9,10,11]; and elderly people with a weakened state of life [12].

The present manuscript is a tentative attempt to extend the scope of nutritional expertise acquired in Westernized societies to those developing countries trying hard to prevent the occurrence of dietary deficiencies and to improve the nutritional value of their customary regimens. This proposal is priorly addressed to those underprivileged children and women living under poor sanitary conditions. At least two lines of approach deserve to be explored: the *first* would direct principal attention to the usual proportion of protein and amino acid content found in regular dishes and the *second* would set forth more efficient laboratory tools among those already available to follow-up the global health state of enrolled subjects.

## 2. Biomarkers Pertaining to the PINI Formula

Table 1 collects the main physicochemical characteristics of the four biomarkers implicated in the PINI formula in both sexes, all age classes, and morbidity classes. Both CRP and AGP indices were strongly recommended by the WHO [13,14] and are widely used nowadays by working teams engaged to screen underprivileged inhabitants in developing countries, allowing them to quantify their acute or chronic inflammatory disorders, respectively. The below review provides some recent biological findings strengthening the validity of the PINI formula in field studies.

### 2.1. Albumin (ALB)

ALB was first described by Ancell as a component of animal body fluids allowing the prevention of edema [15]. ALB is a simple and unglycosylated monopeptide chain comprising 35 cysteine (Cys) amino acids (AAs) wherein 34 are involved in 17 intramolecular S–S bridges with a single Cys34 residue remaining in the free and redox-active form. ALB molecules exhibit a tertiary isomeric conformation. The gene coding for ALB is on the long arm of chromosome 4. The intravascular space sequesters about 30–40% of total body ALB [16] and the remainder component is primarily in extracellular fluids, including cerebrospinal fluid (CSF). Its concentration in lymphatic fluid approaches that of plasma [17]. ALB is mainly synthesized by the liver depending on the number of AAs in customary diets [18]. It is indeed shown that protein restriction specifically decreases the abundance of albumin nuclear transcripts and mRNA levels [18]. Plasma ALB contributes to approximately 80% of the total colloid osmotic pressure, binds and transports several hormones, metabolites, and drugs (AAs, vitamins, fatty acids, phospholipids, micronutrients, and unconjugated bilirubin). ALB is one of the three specific carrier proteins for both thyroid hormones [19], constituting a reservoir of free AAs available to meet tissue requirements [20]. ALB is also vitally important as an anti-oxidative factor representing more than 70% of plasma free-radical trapping activity, working mainly during inflammatory conditions through its multiple binding sites and scavenging properties [21]. Recent animal studies defend the view that ALB molecules may develop proper redox potential as a result of protein nutrition imbalance, hence manifesting ambivalent pro- and con-oxidative capacities [22].

The proximal tubular uptake of ALB is extremely efficient, as shown by the statement that in healthy adults, the high liver production rate of ALB (~20 g/day,) contrasts with trace amounts of urinary losses not exceeding ~30 mg/day, thus pointing to an ALB-sieving coefficient of 0.00062 [23]. The ALB resorptive processes stand under the control of megalin and cubilin, two receptors belonging to the proximal tubules [24]. The data show that, under steady state conditions, kidneys maintain important regulatory roles in the metabolic equipoise between protein intake and protein output. After glomerular filtration, ALB molecules are taken up into lysosomes of renal tissues within 6 to 15 min and undergo decay after 30 to 120 min followed by recycling into AA pathways [25]. Cytokine-induced inflammatory disorders may cause an acquired dysfunction of megalin and cubilin activities marked by impaired re-uptake of ALB and ensuing albuminuria [24]. This distortion from normal appears to be aggravated by the surge of AGP [26] causing disruption of the glycocalyx/endothelial surface layer together with functional alterations of podocyte activities [27]. Elevated albuminuria may also be causally related to liver damage, transcapillary leakage, and breakdown [21]. On admission, patients with 30–31 g ALB/L incur increasing risks of clinical complications [28]. Values lower than 30 g/L augment the likelihood of a prolonged hospital stay and infectious complications at the site of injury [29]. Chinese workers have shown that the lower limit compatible with survival in severe sepsis is 24.5 g ALB/L [30]. Taken together, these data indicate that ALB values working alone may constitute warning signals and predictive indicators of morbidity and mortality outcomes. The measurement of ALB as a nutritional biomarker was published after electrophoretic analysis of serum from protein-calorie malnourished (PCM) children [31]. The method readily became a widely accepted analyte conducive to worldwide expansion in most clinical laboratories during the following two decades [32].

### 2.2. Transthyretin (TTR)

TTR plasma protein (previously named prealbumin) is one of the three carrier-proteins of both thyroid hormones [19]. Its fascinating property to function as a nutritional biomarker was established on the occasion of a pediatric project undertaken at the University of Dakar (Senegal) aiming at developing comprehensive investigations on all aspects of thyroid dysfunction in PCM children [33]. The full study lasted seven years and the results were collected in a dissertation that was successfully defended at the Catholic University of Louvain (Belgium) in 1977 to obtain the PhD degree [34]. By comparison with ALB, until then regarded as a gold standard, TTR did reveal steeper slopes indicating a higher level of sensitivity to protein depletion and repletion [33]. The distinct behavior of TTR was mainly attributed to a shorter T_1/2_ allowing the early detection of marginal PCM as well as the reliable follow-up of any severe nutritional disorder [33]. The tetrameric TTR edifice possesses one monomeric subunit bearing a binding site for one molecule of retinol-binding protein (RBP, unglycosylated monopeptide sequence of 182 AAs, 21 kDa as MM) revealing in turn an inner cleft harboring a single molecule of retinol, thereby forming the holo-RBP molecule [35]. Agglomeration of holo-RBP to TTR occurs in the liver parenchymal cells before its exportation into the bloodstream in the form of a retinol circulating complex (RCC, 76 kDa as MM) [36]. Despite the fact that the biological T_1/2_ of holoRBP is four times more rapid than that of TTR (half a day vs. 2 days), the three RCC components remain attached at a close 1:1:1 stoichiometry [36], showing that TTR is the main determinant and the limiting factor ensuring the bioavailability of the RBP–retinol dyad to target tissues.

In recent years, major breakthroughs were attained by the confrontation of two distinct and huge clinical investigations running in parallel to highlight some basic aspects of our current nutritional knowledge. The first consists of acknowledging the pioneer investigations performed under the expert guidance of G.B. Forbes [37] at the University of Rochester (New York, NY, USA, 14642). The technical assistance of the International Atomic Energy Agency (IAEA, Vienna, Austria) requiring dual-energy X-ray absorptiometry (DXA) methodology was used for measuring the naturally occurring radioisotope ^40^K confined within all body cells in combination with nonradioactive ^39^K. In body tissues of healthy subjects, the distribution of both K elements maintains their tight relationships with nitrogen (N) [38], thereby allowing researchers to collect accurate information on the individual content of N in each body compartment regardless of age and gender and to assess the global accretion of N in lean body mass (LBM). The second innovative thrust was performed by J. Bienvenu et al. [39] aiming at measuring TTR values of 68,720 healthy US citizens from birth to very old age using Beckman reagents, turbidimetric equipment (Brea, CA, USA, 92622), and the technical support of the Foundation for Blood Research (Scarborough, ME, USA, 04070). This very large survey has exacted many years hardworking before attaining completion. Results, means, and standard deviations (SD) are published elsewhere [40], showing that both LBM data, recorded by using DXA technology and immunologic assessment of TTR values, disclose similar evolutionary patterns in the process of time together with maintenance of highly positive correlations during the entire human lifespan [41,42]. Moreover, occurrence of any morbid disorder, whatever its causal factor, indicates that TTR remains a valid and reliable nutritional biomarker of LBM in health as well as in any disease process. A recent prospective study using a receiver operating characteristic (ROC) curve of TTR values measured in adult subjects has yielded a range from 170 mg/L to 120 mg/L for upper and lower cut-off lines, respectively [43]. The data are consistent with current clinical practice pointing to 200 mg/L and 100 mg/L for the upper and lower limits of the grey zone separating normality from lethality [42]. In PCM children, the lower TTR concentration compatible with survival is defined at 65 mg/L [44].

### 2.3. C-Reactive Protein (CRP)

This APP was discovered in 1930 [45] displaying a nonglycosylated pentameric conformation composed of five identical subunits arranged with cyclic symmetry. Its denomination derives from the initial observation that CRP was able to bind and precipitate the somatic C-polysaccharide fraction of pneumococci. The CRP gene is located on the proximal long arm of chromosome 1 between band q12 and band q23 [46]. CRP is secreted by the liver and circulates in the bloodstream at very low concentrations under healthy conditions (≤1 mg/L) but may rapidly increase up to a hundredfold during the acute stage of inflammatory processes with peak values reached 2–3 days later [47]. CRP does not cross the placental barrier but is synthesized during fetal life and by newborn infants. CRP preferentially recognizes Gram + bacteria; some parasitic agents and fungi; and neoplastic, necrotic tissues, and cell debris, thus playing a major role in the early phase of stressful disorders as a potent stimulator of the complement cascade, activating the phagocytosis of microorganisms by plasma leucocytes [48,49].

### 2.4. Alpha-_1_ Acid Glycoprotein (AGP)

AGP is one of the most glycosylated plasma proteins belonging to the lipocalin family that was isolated and described by Weimer in 1950 [50]. Its linear monopeptide sequence comprises 183 AAs bearing 5 glycosylated antennae through N-linkage to asparagine (Asn) in positions 15, 38, 54, 75, and 85 [51]. Human AGP is encoded by three genes clustered in one locus mapped on the distal portion (band q34) of the long arm of chromosome 9 [52]. The MM of AGP is about 41 kDa with a carbohydrate moiety of approximating 42% depending upon the proportion of glycan sites that may undergo varying diminished or enlarged structural alterations having functional significance. The liver production of AGP is low at birth but rises gradually from the first week of life to reach adult levels by about 1 year [53]. The liver synthesis of AGP is poorly influenced by changes in several types of dietary regimens [54]. AGP comports as an APP stimulated by Il_-1_, Il_-6_, and steroid compounds [55], yielding concentrations subjected to large fluctuations in disease processes. AGP levels participate in several immune processes [56], sepsis [57], malarial virulence [58], viral contamination [59], and parasitic infestation [60] but also in hormonal, vascular, drug transport, and tissue distribution. The modulatory binding to antennae of sialylated variants and fucosyl residues leads to polyvalent microheterogeneity. Laboratory measurements of these AGP conformational changes appear to be helpful to disentangle the complex pathophysiology of several morbid processes, notably those generated by hepatic disorders [61,62]. AGP is used in developing countries as a helpful analyte to detect and follow up the outcome of most chronic and long-lasting inflammatory events, reaching fluctuating levels ranging up to fivefold of the baseline values. Following the demonstration that AGP provides early sepsis responses [57], a research group of Cornell University (Ithaca, NY, USA, 14853) developed a simple lateral flow immunoassay allowing the quantification of AGP using rapid and transportable equipment ideally suited to fill screening programs in developing countries [63].

## 3. Discussion

The PINI scoring system is a biochemical tool described nearly four decades ago and constitutes a ratio between two products. The numerator aggregates CRP and AGP, which were selected from seven candidates worthy of being chosen as the best and complementary indicators of inflammatory burden. Both biomarkers were initially dedicated to screening adult sick patients in hospital settings [1] but went readily beyond this spectrum to identify infectious problems inflicting children and women living under poor sanitary conditions in developing countries. Dozens of such studies were undertaken during the last two decades, mainly in Africa and Asia, supported by the strong recommendations of the WHO [13,14] and humanitarian agencies. This technical strategy has nowadays gained worldwide recognition.

The denominator of the PINI formula consists of the product of two liver biomarkers assessing the overall protein nutritional status of recruited subjects. The uniqueness of TTR lies in the fact that it is the sole plasma protein maintaining closely correlated relationships with LBM from birth to very old age, whatever the gender or disease state [41,42]. In any type of inflammatory disorder characterized by cytokine-induced urinary N-losses from LBM organs, TTR is endowed with the unique responsibility to quantify the proportion of LBM undergoing N-depletion during the inflammatory process while pinpointing the fraction remaining available for tissue immune and repair responses. Working alone, TTR is indeed able to assess the protein nutritional status of growing neonates [64] and of children with solid tumors [65]; to evaluate the outcome of declining elderly subjects [66] and of critically ill subjects [67]; and to appraise the current health state of burned [68], surgical [69], renal [70], cardiac [71], neurosurgical [72], COVID-19 [73], acute stroke [74], and Alzheimer patients [75].

The intravascular volume of normal adults contains a mean TTR value ~300 mg/L whereas that of ALB is ~40 g/L, meaning a molar TTR: ALB ratio of less than 1:100. Together with this unequal poise, both TTR and ALB biomarkers demonstrate distinct and complementary kinetics [76,77,78] revealing several readjusting steps with LBM tissues. The small concentration of TTR does not preclude this index from playing the informative role as the best indicator of LBM stores in the whole body with the highest known correlation coefficient r reaching 0.64 [79] whereas the ALB concentration is distinguished by a significantly lower but not negligible r value of 0.52 [79]. It is of interest to take note that in healthy individuals, the urinary loss of TTR is extremely low, not exceeding 150 μg/L while remaining highly correlated with albuminuria (r = 0.85) [80]. The largely predominant plasma ALB concentration has the additional advantage of buffering the sometimes unusually elevated CRP values, hence helping to stabilize the whole PINI scoring formula.

### 3.1. The RBP–Retinol Binary

Retinol is provided to the human body through the diet and is sequestered in hepatic stellate cells after its hydrolysis as retinyl esters. The liver constitutes the main storage organ of VA that may be exported following tissue requirements provided that de novo TTR molecules be available. After RCC transportation to target tissues, holoRBP releases its ligand to membrane and cytosolic receptors to become apoRBP, a catabolic derivative characterized by a sequence of 181 AAs, devoid of a terminal Asn residue and marked by a shortened T_1/2_ of 3.5 h [81]. ApoRBP molecules undergo rapid kidney uptake and proximal tubular recycling explaining that they are not recovered in the urinary output of healthy individuals. This metabolic cycle has long sustained the view that the measurement of RBP and/or retinol values in the bloodstream might provide equally relevant information concerning the VA status of the body [13]. The choice of RBP is usually the preferred option, owing to easiest and inexpensive immunoassays contrasting with either the quantitative measurement of liver vitamin A using dose–response tests (DRT) [82] or the retinol isotope dilution test (RID) requiring [^13^C]retinyl acetate [83].

**Figure 1:**
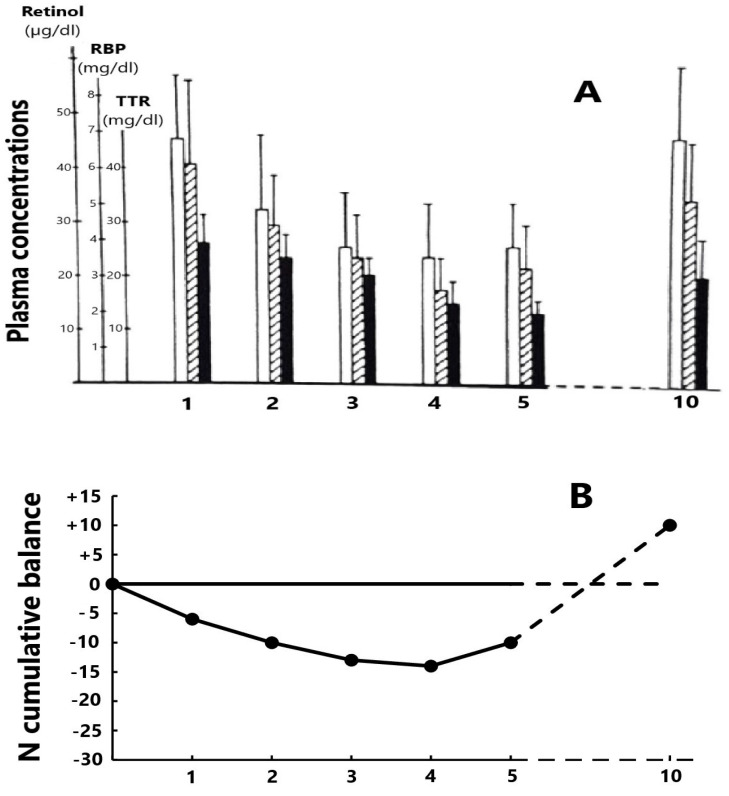
Evolutionary patterns of the RCC edifice during a depletion period following a stress of medium severity. (**A**) The 3 components of the RCC measured in 15 healthy adult hysterectomized females [84] maintain close relationships during the post-operative period (□ retinol, 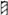 RBP and ■ TTR). The nadir level is reached on days 4–5 and, in the absence of complications, healing is recorded on day 10 with full restoration of basal RCC values. (**B**) The cumulative mean N balance in 28 healthy adult subjects undergoing abdominal surgery and who were enterally refed [85]. Urinary excretion of nitrogenous compounds is mainly due to the cytokine-induced breakdown of tissue proteins stored in lean body mass (LBM) whose depletion may be quantified by TTR values showing the same declining profile as that of N.

Former studies have appraised the trajectory of the RCC edifice in patients enduring an acute episode of a surgical or inflammatory disorder. Such stressful circumstances are marked by cytokine-induced abrogation of TTR synthesis dragging down the two other RCC components and maintaining unaltered relationships [84] (Figure 1A). Cytokines also generate LBM proteolysis in the whole body leading to increased urinary losses of nitrogenous catabolites with a N balance turning negative to reach a nadir level by day four or five (Figure 1B) and are hence associated with disclosure by the RCC triad [85]. The dietary rehabilitation of PCM children initiates the gradual restoration of TTR–RBP–retinol values toward normal levels, keeping unmodified RCC relationships [86] (Table 2). The depletion RCC curve is primarily determined by the surge of cytokines suppressing the production of liver TTR [87,88], whereas the refeeding RCC curve is principally stimulated by de novo synthesis of TTR releasing holoRBP molecules [18,89]. As a result, the transient decrease of RCC values in both inflammatory and nutritional disorders appears to be unrelated to the liver retinoid stores. It is nevertheless worth noting that the stoichiometric relationships between the three RCC molecules are more stable in refeeding studies (Table 2) than in depleting processes (Figure 1A) owing to the more versatile behavior of RBP during inflammatory conditions.

It is necessary to establish further insight and to focus more attention on the roles played by RBP and retinol, regarded as major biomarkers of VA status in field studies. At least three salient domains exist and undermine the reliability of RBP–retinol indices in VA deficiency. *First*, current anti-RBP immunoassays do not differentiate holoRBP from apoRBP molecular species [90]. This peculiarity also concerns novel post-translationally processed forms of RBP, one of which (devoid of two terminal leucine [Leu] residues) is shown markedly elevated in chronic renal failure [91]. Combined measurement of such heterogeneous molecules have distinct functional activities and explain the unusually large RBP plasma distribution range which depreciates the relevancy of statistical comparisons between means, medians, and standard deviation values. *Second*, all febrile infections cause kidney dysfunction and the leakage of low MW proteins, among which α_1_-microglobulin (26 kDa), β_2_-microglobulin (11.8 kDa), and RBP (21 kDa) are the most frequently studied. In that context, RBP appears to be a very sensitive and reliable marker of early and declared proximal tubular injury [92]. A large investigation comprising 186 kidney patients submitted to renal biopsy did demonstrate that the specificity and sensitivity for renal tubular damage of RBP were 91.03% and 72.06%, respectively [93]. Animal experiments have shown that a dysfunction of the tubular megalin receptor downregulates the re-uptake of both RBP and retinol molecules by renal tissues, hence increasing their urinary leakage in the free form [94]. Similarly, the augmented kidney wastage of RBP and retinol is currently recorded in human subjects submitted to inflammatory disorders [95,96]. In the case of ALB, besides the above-cited detrimental role played by AGP [26], it appears that cytokine-induced functional defects of tubular megalin and cubulin contribute to albuminuria losses [24]. *Third*, it is worth mentioning that the correlation coefficient r linking RBP to LBM resources is 0.57 [79], thus holding an intermediate position higher than ALB but lower than TTR.

The recorded data show that RBP should no longer be regarded as a biomarker of VA status owing to the diversity of plasma carrier-proteins, receptors, and retinoid derivatives in liver, body tissues, and urinary output [97,98], each disclosing up and down fluctuations according to specific metabolic impulses. This proposal is supported by clinical investigations undertaken in infected PCM children [99,100], in kidney patients [101], and in COVID-19 vaccination contexts [102]. It seems very unlikely that RBP, working alone, might encompass such varying evolutionary patterns. Most attempts to set up adjusted procedures aimed at improving inconsistent results found in field studies failed to succeed with evidence of substantial biases. A more realistic approach should take place, serving as a base for the trustworthy RCC data recorded in both stressful disorders (Figure 1A) [84] and dietary rehabilitation (Table 2) [86], showing that RBP is nothing other than a TTR-mediated surrogate biomarker of LBM. The significant difference between RBP and TTR data lies in the fact that the latter analyte remains confined inside the intravascular space and neither undergoes meaningful extravasation nor urinary leakage. The withdrawal of RBP and its replacement by TTR for the evaluation of VA status should allow ALB to occupy the second correlation rank with LBM, thereby forming with TTR a bipolar association able to provide a strong estimate of global protein status. This proposal is grounded on the view that optimal health status depends on full LBM replenishment, implying normalization of ALB and TTR plasma concentrations. Such approaches should trigger substantial gains in nutritional knowledge, paving the way for renovated models of relationships between VA and N reserves.

### 3.2. The Iron-Deficient Anemia Burden

Fe-deprived anemia (IDA) is a major scourge inflicting very large population groups worldwide, being held by WHO experts as a main nutritional disorder needing to be tackled priorly to improve the state of health of mankind [14]. According to WHO reports, the world prevalence of ID indicates that nearly half of all children under five years of age are anemic and/or Fe-deficient [103].

Taken together, it is possible to schematically distinguish three main groups of factors causally involved in the occurrence of ID. *First*, those generated in large population groups living in poor sanitary and low-income conditions, mainly in desertic areas, are chronically deprived of the basic intake of nutrients required to ensure normal erythropoiesis (proteins, folates, cobalamins, carotenoids, and zinc). Their situation may be dramatically aggravated with the advent of drought, reduced crops, civil wars, and population displacements. *Second*, ID can result from the parasitic contamination of human beings living in several African and South American countries, such as the intestinal tract of exposed subjects being infested by *Ankylostoma duodenale* and *Necator americanus* or infection by *Schistosoma haematobium* which usually predates kidney and bladder tissues leading to a net body loss of Fe through the mediation of fecal or urinary bleeding, respectively. *Third*, ID affected by acute or chronic inflammatory disorders combined with varying levels of nutrient deficits specifically targets preschool children and adult women of developing countries, resulting in a vast number of diseased persons weakened by these interacting factors. Following the BRINDA (Biomarkers Reflecting the Inflammatory and Nutritional Determinants of Anemia) project, the planetary distribution of ID people is characterized by a low, medium, or high infectious burden with the top incidence being reached in several Asian and African countries [104].

Fe homeostasis within the human body comprises dynamic and highly sophisticated interactions between several body proteins, urging the selection of informative data to provide some comments which might be helpful to workers engaged in field surveys. In the absence of intestinal bleeding caused by hookworm infestation, the fecal loss of Fe is proportionate to its abundance in customary regimens. In adult individuals, the dietary requirement of Fe usually ranges from 15 to 30 mg/day with large variations following regional dietary habits. The highest intestinal Fe absorption reaches about 30% with products containing heme molecules (meat and fish) contrasting with a lower intake of 10–15% with items comprising nonheme compounds (dairy, eggs, cereals, and legumes). This means that only a minor fraction of dietary Fe is embodied into biological processes whereas the largest proportion remains unabsorbed in the luminal tract. The urinary loss of Fe is very low in healthy people, ranging from 3 to 10 μg/day [105] but this basal level may nevertheless augment in the course of tubular nephropathies [106] given that each transferrin molecule carries two Fe ions in ferric form. This kidney Fe leakage appears to be swept away by the increased urinary output of TF [107] that remains highly correlated with ALB excretion rate (r = 0.97), highlighting that both biomarkers display closely comparable MM.

The endogenous metabolism of Fe follows some interacting steps that are hereunder briefly summarized for the sake of getting a condensed overview of Fe homeostatic mechanisms. *Transferrin* (TF) is a β-globulin (79.6 kDa as MM) produced by the liver consisting of a polypeptide chain of 678 AA residues and two Fe-binding sites [108]. Following body requirements, the principal roles played by TF consist of the uptake of Fe from enterocytes, its carriage into the bloodstream, and its delivery to target cells, mainly erythropoietic tissues. In healthy subjects, the TF plasma concentration ranges from 2 to 4 g/L. Its usual saturation level is approximately one-third (~100 μg/L) of its maximal binding capacity (~360 μg/L). *Ferroportin* (protomeric sequence comprising 571 AAs with many different variants) is a cellular protein detected at the surface of duodenal enterocytes and playing key roles in the absorption of dietary Fe. It is also implicated in the storage and exportation of Fe from hepatocytes and macrophages [109]. *Hepcidin* is a small molecule (sequence of 25 AAs and 279 Da as MM) secreted by hepatocytes that bind directly to ferroportin on the cellular surfaces of enterocytes, macrophages, and hepatocytes [110]. The data show that hepcidin and ferroportin are interconnected in a story of complex and balanced recycling for the regulation of Fe homeostasis [111]. In stressful disorders, cytokines (mainly Il-_6_) stimulate hepcidin synthesis causing the disappearance of ferroportin from cell membranes, thereby inhibiting the absorption of intestinal Fe as well as its efflux into plasma. As a result, depressed TF saturation and impaired erythropoiesis may occur, leading to the development of ID anemias typically observed in chronic inflammation [111,112]. Conversely, the overproduction of ferroportin may downregulate hepcidin synthesis and induce the reversal of all above metabolic patterns, entailing massive Fe overload in exposed subjects and to the risk of hemochromatosis [113]. *Ferritin* (SF) is a cytosolic iron-storage protein that works as a buffer between Fe deficiency and Fe overload. It is a globular protein comprising 24 subunits (474 kDa as MM) found in most body tissues [114] and forming an intracellular pool of about 3–4 g in human adults, meaning about 35–50 mg Fe per Kg/body weight. These endogenous stores release small proportions of measurable SF in plasma regarded as reflecting the amount of Fe sequestered in the whole body.

## 4. Diagnostic Tools and Dietary Strategies

An overall agreement that the measurement of plasma CRP and AGP identifies both (sub)acute and chronic evolutionary facets of any inflammatory disorder exists [1]. These statements, endorsed by WHO authorities [13,14], are confirmed by the best experts involved in field studies, notably those undertaken by BRINDA and MINDI (Multiple Infections, Nutrient Deficiencies, and Inflammation) research teams [60,115,116]. During the last two decades, those workers have provided a tremendously high volume of scientific data helping to clarify the multiplicity of factors causing dietary deficiencies in populations living in developing countries. The aim of the present section is not to provide extensive comments on these studies, but to pinpoint some peculiar aspects formulated by a clinical nutritionist having himself experienced 20 years of field studies in many African countries. As a result of the fact that poverty and malnutrition have seriously refrained accessibility to balanced regimens, many attempts were proposed to commercially tackle the challenge and to launch nutritious refeeding products. The ready-to-use therapeutic food (RUTF) is an emblematic example of this policy [117] that has known worldwide dissemination. The RUTF concept did initially consist of a 30% milk powder combined with several plant blends and fortified with micronutrient formulations. In this type of regimen, the milk powder alone accounts for about 29% of the RUTF cost, a level rapidly regarded as too expensive [117]. As a result, many international agencies supporting these rehabilitation projects did exert strong pressures to cut the cost by replacing part of the milk with an increasing proportion of cheaper vegetable ingredients such as rice and maize powder; sesame seed and sweet potato paste; and peanuts, beans, and soy products.

This strategy was financially sustained by official and private funding sources with the help of sophisticated laboratory tools. After early and promising stages, most BRINDA and MINDI projects did progressively impede halfway between expectations and results [118], yielding the delivery of suboptimal micronutrient effects. The occurrence of multiple inconsistencies and unexpected and/or unexplained data did incite many workers to use adjustment factors of RBP [119], of soluble TF receptor (sTfR) [120], and of SF [121] concentrations for inflammation on vitamin A and/or iron status. It is necessary to highlight that RUTF formulations based on cereals and legumes may harbor anti-nutritional compounds. Indeed, it is notably the case for soybean isolates that contain phytic acid and conglycinin (7S), two major inhibitors of Fe-absorption at the enterocyte level [122]. More importantly, plant products do not display the same AA profiles as those characterizing animal tissues, providing hardly half the abundance of N and essential AAs (EAAs) than animal meals on a weight basis. The data are consistent with dietary surveys showing that the usual consumption of essential AAs is reduced by half in strict vegans [123]. As a result, these individuals demonstrate a highly significant plasma drop (≤0.001) in Leu, lysine (Lys), methionine (Met), and tryptophan (Trp) concentrations [123]. Cassava and maize are largely consumed in Africa, ensuring the major provision of energy while being depleted in at least three EAAs: Lys, Met, and Trp. The EAAs plasma concentration of African children living in rural areas is about 10–20% lower than that of their healthy counterparts. The chronic intake of plant-based dietary regimens is clinically identifiable in underprivileged populations by significant growth retardation, shorter height, and shrunken LBM [124]. The issue of imbalanced vegan diets was addressed by the Massachusetts Institute of Technology for children [125] and adult subjects [20,126] that at least one-third of total protein should originate from animal sources. This estimation is regarded as fulfilling the minimal N requirements of healthy persons. The renowned US expert W. Rose, after pioneer studies dedicated to the structure and function of most EAAs, summarized their nutritional needs in the following manner: «You can’t build proteins unless you have all necessary blocks. If you take out one of them, then the rest become largely useless» [127]. It seems therefore mandatory to come back to the proportion of 30% animal proteins found in initial RUTF products. The attainability of such an objective requires milk beverages (the cheapest of all animal sources) [128] or other animal foods [129] to increase the EAA supply with a view to improving the nutrient adequacy of plant products. Such a public health policy has a cost, but it is the sole efficient way to obtain rapid and efficient advances in nutritional rehabilitation programs.

Concerning ID therapy, several approaches are undertaken. Chronic infestation by hookworms of the intestinal tract or kidney excretory ducts may lead to substantial net losses of Fe in fecal and urinary outputs. Orderly deworming and forceful therapy with Fe molecules are thereby obligatory. Blood transfusions may be required to improve the most severe Fe-depleted states. A recently published Ethiopian paper has provided a comprehensive review on the socio-economic background lying behind the public health problem of anemia in the whole African continent [130]. The pooled prevalence of anemia among under-five children is 59% and the main determinants are the female sex, maternal education, residence, and family size. The lowest prevalence [7%] was observed in Rwanda and is likely explained by the unfit proliferation for mosquitoes in mountainous highlands [131]. The highest prevalence [87%] was recorded in the coastal and midlands areas of Senegal [132]. African and Asian global pool prevalence of IDA and ID demonstrates a similar impact among children under the age of five, reaching 16.4% IDA and 17.9 ID, respectively [133]. According to the infectious environment and dietary customs, significant differences are reported between countries: children living in the Democratic Republic of Congo account for 46.6% of IDA and 16.5% of ID [134] whereas those dwelling in Burkina Faso amount to 42% of IDA and 50% of ID [135]. The dietary prescriptions and nutrient premix provided to the cohorts of children and women hospitalized for the assessment and treatment of VA and Fe-deficits display results approaching those already above-described. In most cases, some limited beneficial effects are recorded without reaching normalization, thus remaining suboptimal. Some data deserve to be underlined, notably the up and down disruption of the retinol/RBP molar ratio (≥1 and ≤0.8) in 34% and 44% of Panamanian lactating women, respectively [136], hence confirming the uncertainty attributed to RBP as an indicator of VA status [99,100,101,102]. In Burkina Faso, most indices (RBP, Hb, and SF) measured at discharge showed that the improvement of VA states is associated with a significant lowering of sTfR [135] usually regarded as a marker of hematopoiesis intensity. A possible explanation lies in the fact that the production of TF is depressed in PCM states similarly to other nutritional biomarkers (ALB, TTR, and RBP) [137] but is strongly stimulated by IDA, indicating that the hepatic synthesis of TF may be subjected to opposite stimuli [138].

The diagnostic and prognostic follow-up of these nutritional and inflammatory disorders is best insured by the combined measurement of the four objective indices gathered within the PINI scoring system described nearly four decades ago [1]. The binary CRP–AGP association makes it possible to monitor acute and chronic infectious diseases and gives a driving force to a whole range of field studies worldwide [60,115,116]. The binary TTR–ALB nutritional component of the PINI formula benefits from close correlations with LBM values (0.64 and 0.52), respectively [79]. LBM indeed collects all organs and tissues harboring the bulk of all N-containing organs, therefore assuming the role of a cornerstone of bodybuilding [40,41]. Optimal health is a physiological state generated by full N-replenishment of all bodily pools forming LBM together. This last condition coincides with the normalization of plasma TTR values that may be therefore regarded as the most useful nutritional marker showing the nearest LBM proximity and exquisitely sensitive responses to any LBM fluctuation [40,41]. ALB is less informative, reacting more slowly and indicating long-lasting nutritional tendencies [76,77,78]. These data show that both nutritional biomarkers share in common kinetic similarities with inflammatory indices. Mimicking the example shown by CRP, TTR may be regarded as an early responder to any protein shortage whereas ALB and likewise AGP, demonstrate slackened reactions. Both TTR and ALB analytes nevertheless together form a stable and robust bipolar combination. RBP has long maintained a candidacy position that is now undergoing progressive fading owing to its versatile behavior, notably in VA-deficient states [99,100,101,102].

## 5. What Lesson Is to Be Learnt from the TTR Story?

The story of TTR is narrowly associated with the development of nutritional knowledge, particularly within the decadal debate that has divided the defenders [139] and detractors [140] of protein requirements in global human health. A recently published comprehensive and well-balanced review by R. Semba [141] highlighted most steps of this controversy. McLaren indeed expressed provocative opinions in the columns of The Lancet in 1974, claiming that the rehabilitation campaigns undertaken to restore PCM states in developing countries were a «great protein fiasco» [140]. McLaren’s views have generated substantial dissent among scientific, humanitarian, and political authorities, entailing during years disturbances of the nutritional strategies provided to underprivileged populations. This dramatic episode is nowadays largely obliterated, but remnants of McLaren’s mistaken beliefs persist in the mind of some decision makers, especially when a clear preference for inappropriate and low-cost nutrients is imposed using strong economic pressures prevailing over sound scientific considerations.

The story did start in 1933 when the pediatrician Cecily Williams who was working in the Republic of Ghana (previously named Gold Coast) made the observation that a disease locally named «kwashiorkor» might be induced by maize diets given to weaning children [142]. The Ghanaian denomination was readily adopted by the WHO and the ensuing investigations did reveal that most African countries were inflicted by the same nutritional disorder with the exception of livestock areas producing cows’ milk [143]. The first measurement of plasma ALB was performed in 1951 in South Africa [31] and its application, shown to be useful for the follow-up of KW children, has known a rapid and widespread diffusion worldwide. The first proposal of TTR as a biomarker of protein-depleted states was published in 1972 [33] in a nutritional context described with some details elsewhere [42]. The uniqueness of TTR lies in the fact that it displays the nearest proximity (r = 0.64) with LBM, sticking to its evolutionary patterns from birth to very old age, whatever the gender, age, or disease state [40,41]. In any type of inflammatory disorder characterized by cytokine-induced urinary N-losses from LBM organs, TTR identifies the proportion of LBM tissues remaining available for immune and repair responses. In addition, TTR forms a robust nutritional component of the PINI scoring system with ALB [1]. It is quite puzzling that the Directory Board of the American Society for Parenteral and Enteral Nutrition (ASPEN) has delivered a deluge of so-called position papers for many years [144], aiming at withdrawing TTR and ALB from the hands of clinical nutritionists. This ostracizing policy has generated suspicions among most workers who are now currently using all available nutritional biomarkers, with the sole exception of TTR. Most US clinical laboratories show zealous obedience and conform to the imposed ASPEN recommendations with harmful consequences for the patients who are deprived of the best nutritional coverage. This reluctant attitude has also promoted many critical comments, sometimes expressed with irony or even with a touch of mockery [145]. In contrast, Eastern working teams have readily adopted the novel clinical perspectives offered by TTR. A recent review paper [42] has provided a short inventory of these trends sustaining the view that most Asian countries, mainly China [146], South Korea [147], and Japan [148] have embraced TTR as a reliable and universal biomarker in most medical disciplines. TTR obviously occupies a central position in the battlefield severing the Western hemisphere adhering to obsolescent nutritional concepts that are distinct from the options selected by Eastern countries giving support to innovative strategies.

## Figures and Tables

**Table 1:** Main physicochemical characteristics of biomarkers composing the pini formula.

	Normal Adult Values	Molecular Conformation	Molecular Mass (MM)	Biological Half-Life (T_1/2_)
ALB	35–45 g/L	unglycosylated monopeptide (580 Aas)	66.5 kDa	19 days
TTR	280–350 mg/L	unglycosylated tetramer (4 × 127 Aas)	55 kDa	2 days
CRP	≤1 mg/L	cyclic pentaxin (5 × 206 Aas)	118 kDa	19 h
AGP	0.4–1.4 g/L	monopeptide (183 Aas) 42% glycosylated	41 kDa	60–120 h

**Table 2:** Evolutionary patterns of the RCC edifice during the refeeding program of severely protein-depleted children.

	Day 1 (n = 39)	Day 8 (n = 39)	Day 15 (n = 39)	Day 22 (n = 39)	Day 45 (n= 15)	Day 60 (n= 15)
Retinol	11.62 ± 4.31	25.93 ± 9.99	37.86 ± 9.60	42.35 ± 9.88	39.97 ± 7.79	40.05 ± 7.21
(μg/100 mL)	(2.1–19.3)	(9.9–44.7)	(15.2–54.3)	(32.7–56.6)	(29.9–51.3)	(30.4–50.3)
RBP	1.62 ± 0.71	3.57 ± 131	4.96 ± 1.59	5.43 ± 0.88	5.21 ± 1.01	5.17 ± 1.02
(mg/100 mL)	(0.3–3.5)	(1.5–6.1)	(2.0–8.5)	(4.3–7.6)	(4.4–8.2)	(4.1–7.9)
TTR	6.45 ± 1.86	13.03 ± 4.05	19.78 ± 5.25	22.23 ± 3.24	21.72 ± 3.44	21.63 ± 3.56
(mg/100 mL)	(3.4–11.5)	(7.2–22.6)	(9.6–32.0)	(17.1–29.8)	(18.2–29.9)	(17.6–28.1)

These 39 severely malnourished children were admitted to the pediatric intensive rehabilitation unit. They presented all the typical symptoms of kwashiorkor with conscious alterations, great vulnerability to infectious complications, fatty liver, protracted diarrhea, swollen limbs, and skin lesions. Their mean starting TTR plasma value was 6.45 mg/dL, having ominous prognostic significance [44]. During the first days after hospitalization, they were progressively refed using bovine milk hydrolysate (Nesmida, Nestlé) providing oligopeptides devoid of lactose with no addition of sucrose owing to the usual defective activities of intestinal disaccharidases. The main source of carbohydrate energy was given in the form of glucose. The mean values of the 3 components of RCC were doubled after 1 week and tripled after 2 weeks of dietary rehabilitation, and thereafter maintained at a plateau level until day 60 [86]. All children recovered safely.

## Data Availability

This scientific information is largely available in PubMed and in the US National Institute of Health (NIH) archives.

## Appendix A

PINI FORMULA

$$\mathrm{PINI}=\frac{\alpha_{1}\mathrm{-AGP}\;(\mathrm{mg}/L)\;\times \;\mathrm{CRP}\;(\mathrm{mg}/L)}{\mathrm{ALB}\;(g/L)\;\times \;\mathrm{TTR}\;(\mathrm{mg}/L)}$$

STRATIFIED SCALING OF GHS VALUES

≥40: life-threatening state
31 to 40: severe GHS state
21 to 30: medium impact GHS state
11 to 20: moderate impact
1 to 10: low impact
≤0: steady state

Taken as a whole, the PINI formula provides a balanced evaluation of the results yielded by each of both inflammatory and nutritional components, thus allowing the calculation of GHS. In healthy subjects, GHS results are neutral and close to 0, but they rise progressively as the infectious burden tends to aggravate and/or the nutritional deterioration worsens. Each component of the formula evolves for its own account, both giving the clinician the capacity to focus more specific attention on the component showing increasing frailty and requiring a more adapted therapeutic strategy. When the morbid process undergoes progressive subsiding, the GHS formula decreases accordingly until the starting neutrality level is recovered.

## References

[1] Ingenbleek Y., Carpentier Y.A. (1985). A prognostic inflammatory and nutritional index scoring critically ill patients. Int. J. Vitam. Nutr. Res..

[2] Pressac M., Vignoli L., Aymard P., Ingenbleek Y. (1990). Usefulness of a prognostic inflammatory and nutritional index in pediatric clinical practice. Clin. Chim. Acta.

[3] Kudlácková M., Anděl M., Hájková H., Nováková J. (1990). Acute phase proteins and prognostic inflammatory and nutritional index (PINI) in moderately burned children aged up to 3 Years. Burns.

[4] Gϋnel E., Cağlayan O., Cağlayan F., Sahin T.K. (1998). Acute-phase changes in children recovering from minor surgery. Pediatr. Surg. Int..

[5] Vehe K.L., Brown R.O., Kuhl D.A., Boucher B., Luther R.W., Kudsk K.A. (1991). The prognostic inflammatory and nutritional index in traumatized patients receiving enteral nutrition support. J. Am. Coll. Nutr..

[6] Schlossmacher P., Hasselmann M., Meyer N., Kara F., Delabranche X., Kummerlen C., Ingenbleek Y. (2002). The prognostic value of nutritional and inflammatory indices in critically ill patients with acute respiratory failure. Clin. Chem. Lab. Med..

[7] Dessi M., Noce A., Agnoli A., De Angelis S., Fuiano L., Tozzo C., Taccone-Gallucci M., Federici G. (2009). The usefulness of the prognostic and nutritional index in a haemodialysis population. Nutr. Metab. Cardiovasc. Dis..

[8] Gharsallah H., Hajjej Z., Naas I., Aouni Z., Stambouli I., Ferjani M. (2014). Assessment of nutritional status and prognosis in surgical intensive care unit: The prognostic and inflammatory index (PINI). Int. J. Nutr. Food Sci..

[9] Walsh D., Mahmoud F., Barna B. (2003). Assessment of nutritional status and prognosis in advanced cancer: Interleukin-6, C-reactive protein, and the prognostic and inflammatory nutritional index. Support Care Cancer.

[10] Dupire S., Wemeau M., Debarri H., Pascal L., Hivert B., Willekens C., Boyle E., Manier S., Thielemens B., Onraed B. (2012). Prognostic value of PINI index in patients with multiple myeloma. Eur. J. Hematol..

[11] Kirov K.M., Xu H.P., Crenn P., Goater P., Tzanis D., Bouhadiba M.T., Abdelhafidh K., Kirova Y.M., Bonvalot S. (2019). Role of nutritional status in the early postoperative prognosis of patients operated for retropperitoneal liposarcoma (RLS): A single center experience. Eur. J. Surg. Oncol..

[12] Bonnefoy M., Ayzac L., Ingenbleek Y., Kostka T., Boisson R.C., Bienvenu J. (1998). Usefulness of the prognostic inflammatory and nutritional index (PINI) in hospitalized elderly patients. Int. J. Vitam. Nutr. Res..

[13] WHO (2011). Serum Retinol Concentrations for Determining the Prevalence of Vitamin A Deficiency in Populations.

[14] WHO (2011). Serum Ferritin Concentrations for the Assessment of Iron Status and Iron Deficiency in Populations: Vitamin and Mineral Nutrition Information System.

[15] Ancell H. (1839). Course of lectures on the physiology and pathology of the blood and the other animal fluids. Lancet.

[16] Rothschild M.A., Oratz M., Schreiber S.S. (1972). Albumin synthesis. N. Engl. J. Med..

[17] Peters T., Putnam F.W. (1975). Serum Albumin. The Plasma Proteins. Structure, Function and Genetic Control.

[18] Straus D.S., Marten N.W., Hayden J.M., Burke E.J. (1994). Protein restriction specifically decreases the abundance of serum albumin and transthyretin nuclear transcripts in rat liver. J. Nutr..

[19] Robbins J., Harland W.A., Orr J.S. (1975). Structure and function of thyroid transport proteins. Thyroid Hormone Metabolism.

[20] Young V.R., Pellett P.L. (1990). Current concepts concerning indispensable amino acid needs in adults and their implications for international nutrition planning. Food Nutr. Bull..

[21] Soeters P.B., Wolfe R.R., Shenkin A. (2019). Hypoalbuminemia: Pathogenesis and clinical significance. J. Parenter. Enter. Nutr..

[22] Fuka T., Wada Y., Kawakami S., Miyaji K. (2021). Serum albumin redox states: More than oxidative stress biomarker. Antioxidants.

[23] Tojo A., Kinugasa S. (2012). Mechanisms of glomerular filtration and tubular reabsorption. Int. J. Nephrol..

[24] Nielsen R., Christensen E.I., Birn H. (2016). Megalin and cubilin in proximal tubule protein reabsorption: From experimental models to human disease. Kidney Int..

[25] Gekle M. (2005). Renal tubule albumin transport. Annu. Rev. Physiol..

[26] Haraldsson B.S., Johsson E.K., Rippe B. (1992). Glomerular permselectivity is dependent on adequate serum concentrations of orosomucoid. Kidney Int..

[27] Ballerman B.J., Nyström J., Haraldsson B. (2021). The glomerular endothelium restricts albumin filtration. Front. Med..

[28] Viasus D., Garcia-Vidal C., Simonetti A., Manresa F., Dorca J., Gudiol F., Carratalà J. (2013). Prognostic value of serum albumin levels in hospitalized adults with community-acquired pneumonia. J. Infect..

[29] Yamamoto Y., Shigematsu H., Iwata E., Nakajima H., Tanaka M., Okuda A., Kawasaki S., Suga Y., Masuda K., Tanaka Y. (2020). Hypoalbuminemia increased the length of stay in the treatment of postoperative acute surgical site infection in spinal surgery. Spine.

[30] Qian S.Y., Jin D., Chen Z.B., Ye Y.C., Xiang W.W., Ye L.M., Pan J.Y. (2019). Hypoalbuminemia, a novel pronostic factor for prediction of long-term outcomes in critially ill patients with septic shock. Int. J. Clin. Exp. Med..

[31] Anderson C.G., Altmann A. (1951). The electrophoretic serum-protein pattern in malignant malnutrition. Lancet.

[32] Whitehead R.G., Coward W.A., Lunn P.G. (1973). Serum-albumin concentration and the onset of kwashiorkor. Lancet.

[33] Ingenbleek Y., De Visscher M., De Nayer P. (1972). Measurement of prealbumin as index of protein-calorie malnutrition. Lancet.

[34] Ingenbleek Y. (1977). Protein-Calorie Malnutrition in Infants of Young Age. Repercussions on Thyroid Function and Serum Carrier-Proteins. Ph.D. Thesis.

[35] Kanai M., Raz A., Goodman D.S. (1968). Retinol-binding protein: The transport protein for vitamin A in human plasma. J. Clin. Investig..

[36] Monaco H.L., Richardson D.S., Cody V. (2009). The transthyretin-retinol binding protein complex. Recent Advances in Transthyretin Evolution, Structure and Biological Functions.

[37] Forbes G.B. (1987). Human Body Composition: Growth, Aging, Nutrition, and Activity.

[38] Cohn S.H., Vartsky D., Yasumura S., Vaswani A.N., Ellis K.J. (1983). Indexes of cell body mass: Nitrogen versus potassium. Am. J. Physiol..

[39] Bienvenu J., Jeppson J.O., Ingenbleek Y., Ritchie R.F., Navolotskaia O. (1996). Transthyretin & retinol-binding protein. Serum Proteins in Clinical Medicine.

[40] Ingenbleek Y., Richardson D.S., Cody V. (2009). Plasma transthyretin reflects the fluctuations of lean body mass in health and disease. Recent Advances in Transthyretin Evolution, Structure and Biological Functions.

[41] Ingenbleek Y., Bernstein L.H. (2015). Plasma transthyretin as a biomarker of lean body mass and catabolic states. Adv. Nutr..

[42] Ingenbleek Y. (2022). Plasma transthyretin is a nutritional biomarker in human morbidities. Front. Med..

[43] Dellière S.D., Pouga L., Neveux N., Hernvann A., de Bandt J.P., Cynober L. (2021). Assessment of transthyretin cut-off values for a better screening of malnutrition: Retrospective determination and prospective validation. Clin. Nutr..

[44] Dramaix M., Brasseur D., Donnen P., Bawhere P., Porignon D., Tonglet R., Hennart P. (1996). Prognostic indices for mortality of hospitalized children in central Africa. Am. J. Epidemiol..

[45] Tillet W.S., Francis T. (1930). Serological reactions in pneumonia with a non-protein somatic fraction of pneumococcus. J. Exp. Med..

[46] Floyd-Smith G., Whitehead A.S., Colten H.R., Franke U. (1983). The human C-reactive gene (CRP) and serum amyloid P component gene (APCS)) are located on the proximal long arm of chromosome 1. Immunogenetics.

[47] Agrawal A., Kilpatrick J.M., Volanakis J.E., Mackiewicz A., Kushner I., Bauman H. (1993). Structure and function of human C-reactive protein. Acute Phase Proteins.

[48] Whicher J.T., Banks R.E., Thompson D., Evans S.W., Mackiewicz A., Kushner I., Bauman H. (1993). The measurement of acute-phase proteins as disease markers. Acute Phase Proteins.

[49] Baumann H., Gauldie J. (1994). The acute phase response. Immunol. Today.

[50] Weimer H.E., Mehl J.W., Winzler R.J. (1950). Studies on the mucoproteins of human plasma. V. Isolation and characterization of a homogeneous mucoprotein. J. Biol. Chem..

[51] Schmid K., Putnam F.W. (1975). α_1_-Acid glycoprotein. The Plasma Proteins.

[52] Dente L. (1989). Human α_1_-acid glycoprotein genes. Progr. Clin. Biol. Res..

[53] Bienvenu J., Sann L., Bienvenu F., Lahet C., Divry P., Cotte J., Bethenod M. (1981). Laser nephelometry of orosomucoid in serum of newborns: Reference intervals and relation to bacterial infection. Clin. Chem..

[54] Maraj M., Hetwer P., Kuśnierz-Cabala B., Maziarz B., Dumnicka P., Kuźniewski M., Ceranowicz P. (2021). α_1_-Acid glycoprotein and dietary intake in end-stage renal disease patients. Nutrients.

[55] Prowse K.P., Baumann H. (1989). Interleukin-1 and Interleukin-6 stimulate acute phase protein production in primary mouse hepatocytes. J. Leukoc. Biol..

[56] Ceciliani F., Lecchi C. (2019). The immune functions of α_1-_acid glycoprotein. Curr. Protein Pept. Sci..

[57] Xiao K., Su S., Yan P., Han B., Li J., Wang H., Jia Y., Li X., Xie L. (2015). Alpha_1_-acid glycoprotein as a biomarker for the early diagnosis and monitoring the prognosis of sepsis. J. Crit. Care.

[58] Friedman M.J. (1983). Control of malaria virulence by alpha 1-acid glycoprotein (orosomucoid), an acute-phase (inflammatory) reactant. Proc. Natl. Acad. Sci. USA.

[59] Rawat R., Stoltzfus R.J., Ntozini R., Mutasa K., Iliff P.J., Humphrey J.H. (2009). Influence of inflammation as measured by alpha-1-acid glycoprotein on iron status indicators among HIV-positive postpartum Zimbabwean women. Eur. J. Clin. Nutr..

[60] Ayoya M.A., Spiekermann-Brouwer G.M., Stoltzfus R.J., Nemeth E., Habicht J.P., Ganz T., Rawat R., Traoré A.K., Garza C. (2010). Alpha 1-acid glycoprotein, hepcidin, C-reactive protein, and serum ferritin are correlated in anemic schoolchildren with Schistosoma haematobium. Am. J. Clin. Nutr..

[61] Kim S.U., Jeon M.Y., Lim T.S. (2019). Diagnostic performance of serum asialo-α_1_-acid glycoprotein for advanced liver fibrosis or cirhosis in patients with chronic hepatitis B or nonalcoholic fatty liver disease. Korean J. Gastroenterol..

[62] Liang J., Zhu J., Wang M., Singal A.G., Odewole M., Kagan S., Renteria V., Liu S., Parikh N.D., Lubman D.M. (2019). Evaluation of AGP fucosylation as a marker for hepatocellular carcinoma of three different etiologies. Sci. Rep..

[63] Gannon B.M., Glesby M.J., Finkelstein J.L., Raj T., Erickson D., Mehta S. (2019). A point-of-care assay for alpha-1-acid glycoprotein as a diagnostic tool for rapid, mobile-based determination of inflammation. Curr. Res. Biotechnol..

[64] Kim D.H., Lee N.M., Kim S.Y., Yi D.Y., Yun S.W., Chae S.A., Lim I.N. (2021). Effectiveness of prealbumin as an indicator of growth in neonates. Medicine.

[65] Elhasid R., Laor A., Lischinsky S., Postovsky S., Weyl Ben Arush M. (1999). Nutritional status of children with solid tumors. Cancer.

[66] Sato S., Shiozawa M., Nukada S., Iguchi K., Kazama K., Atsumi Y., Numata M., Tamagawa H., Tanaka K., Oshima T. (2021). Preoperative pre-albumin concentration as a predictor of short-term outcomes in elderly patients with colorectal cancer. Anticancer Res..

[67] Devakonda A., George L., Raoof S., Esan A., Saleh A., Bernstein L.H. (2008). Transthyretin as a marker to predict outcome in critically ill patients. Clin. Biochem..

[68] Yang H.T., Yim H., Cho Y.S., Kim D., Hur J., Kim J.H., Lee B.C., Seo D.K., Kim H.S., Chun W. (2012). Prediction of clinical outcomes for massively-burned patients via transthyretin levels in the early postburn period. J. Trauma Acute Care Surg..

[69] Fan Y., Sun Y., Man C., Lang Y. (2021). Preoperative serum prealbumin level and adverse prognosis in patients with hepatocellular carcinoma after hepatectomy: A meta-analysis. Front. Oncol..

[70] Chertow G.M., Goldstein-Fuchs D.J., Lazarus J.M., Kaysen G.A. (2005). Prealbumin, mortality, and cause-specific hospitalization in hemodialysis patients. Kidney Int..

[71] Akashi M., Minami Y., Haruki S., Jujo K., Hagiwara N. (2019). Prognostic implications of prealbumin level on admission in patients with acute heart failure referred to a cardiac intensive care unit. J. Cardiol..

[72] Sugumar D., Arockiaraj J., Amritanand R., David K.S., Krishnan V. (2021). Role of biochemical nutritional parameters as predictors of postoperative morbidity in major spine surgeries. Asian Spine J..

[73] Zinellu A., Mangoni A.A. (2021). Serum prealbumin concentrations, COVID-19 severity, and mortality: A systematic review and meta-analysis. Front. Med..

[74] Isono N., Imamura Y., Ohmura K., Ueda N., Kawabata S., Furuse M., Kuroiwa T. (2017). Transthyretin concentrations in acute stroke patients predict convalescent rehabilitation. J. Stroke Cerebrovasc. Dis..

[75] Ingenbleek Y., Bernstein L.H. (2015). Downsizing of lean body mass is a key determinant of Alzheimer’s disease. J. Alzheimers Dis..

[76] Bernstein L.H., Leukhardt-Fairfield C.J., Pleban W., Rudolph R. (1989). Usefulness of data on albumin and prealbumin concentrations in determining effectiveness of nutritional support. Clin. Chem..

[77] Chertow G.M., Ackert K., Lew N.L., Lazarus J.M., Lowrie E.G. (2000). Prealbumin is as important as albumin in the nutritional assessment of hemodialysis patients. Kidney Int..

[78] Dalrymple L., Johansen K.L., Chertow G.M., Grimes B., Anand S., McCullogh C.E., Kaysen G.A. (2013). Longitudinal measures of serum albumin and prealbumin concentrations in incident dialysis patients: The comprehensive dialysis study. J. Ren. Nutr..

[79] Sergi G., Coin A., Enzi G., Volpato S., Inelmen E.M., Buttarello M., Peloso M., Mulone S., Marin S., Bonometto P. (2006). Role of visceral proteins in detecting malnutrition in the elderly. Eur. J. Clin. Nutr..

[80] Beetham R., Dawnay A., Ghany C., Dubrey S., Miles J. (1993). A radioimmunoassay for human urinary prealbumin. Ann. Clin. Biochem..

[81] Rask L., Vahlquist A., Peterson P.A. (1971). Studies on two physiological forms of the human retinol binding protein differing in vitamin A and arginine content. J. Biol. Chem..

[82] Tanumihardjo S.A., Russell R.M., Stephensen C.B., Gannon B.M., Craft N.E., Haskell M.J., Lietz G., Schultze K., Raiten D.J. (2016). Biomarkers of nutrition for development (BOND)-Vitamin A review. J. Nutr..

[83] Gannon B.M., Tanumihardjo S.A. (2015). Comparisons among equation used for retinol isotope dilution in the assessment of total body stores and total liver reserves. J. Nutr..

[84] Kasper H., Brodersen M., Schedel R. (1975). Concentrations of vitamin A, retinol-binding protein and prealbumin in response to stress. Acta Hepato-Gastroenterol..

[85] Hoover H.C., Ryan J.A., Anderson E.J., Fischer J.E. (1980). Nutritional benefits of immediate postoperative jejunal feeding of an elemental diet. Am. J. Surg..

[86] Ingenbleek Y., Van den Schrieck H.G., De Nayer P., De Visscher M. (1975). The role of retinol-binding protein in protein-calorie malnutrition. Metabolism.

[87] Murakami T., Ohnishi S., Nishiguchi S., Maeda S., Araki S., Shimada K. (1988). Acute-phase response of mRNAs for serum amyloid P component, C-reactive protein and prealbumin (transthyretin) in mouse liver. Biochem. Biophys. Res. Commun..

[88] Banks R.E., Forbes M.A., Storr M., Higginson J., Thompson D., Raynes J., Illingworth J.M., Perren T.J., Selby P.J., Whicher J.T. (1995). The acute phase protein response in patients receiving subcutaneous Il-6. Clin. Exp. Immunol..

[89] de Jong F.A., Schreiber G. (1987). Messenger RNA levels of plasma proteins in rat liver during protein depletion and refeeding. J. Nutr..

[90] Peterson P.A. (1971). Studies on the interaction between prealbumin, retinol-binding protein, and vitamin A. J. Biol. Chem..

[91] Jaconi S., Rose K., Hughes G.J., Saurat J.H., Siegenthaler G. (1995). Characterization of two post-translationally processed forms of human serum retinol-binding protein: Altered ratios in chronic renal failure. J. Lipid. Res..

[92] Bernard A.M., Vyskocil A.A., Mahieu P., Lauwerijs R.R. (1987). Assessment of urinary retinol-biding protein as an index of proximal tubular injury. Clin. Chem..

[93] Xia Y., Peng C.P., Qu S., Liu F., Peng Y. (2011). Correlation studies between urinary retinol binding protein and renal tubular damage. Zhong Nan Da Xue Xue Bao Yi Xue Ban.

[94] Raila J., Willnow T.E., Schweigert F.J. (2005). Megalin-mediated reuptake of retinol in the kidneys of mice is essential for vitamin A homeostasis. J. Nutr..

[95] Ramsden D.B., Princé H.P., Burr W.A., Bradwell A.R., Black E.G., Evans A.E., Hoffenberg R. (1978). The inter-relationship of thyroid hormones, vitamin A and their binding proteins following acute stress. Clin. Endocrinol..

[96] Mitra A.K., Alvarez J.O., Stephensen C.B. (1998). Increased urinary retinol loss in children with severe infections. Lancet.

[97] Gudas L.J. (2022). Retinoid metabolism: New insights. J. Mol. Endocrinol..

[98] Steinhoff J.S., Lass A., Schupp M. (2022). Retinoid homeostasis and beyond: How retinol-binding protein 4 contributes to health and disease. Nutrients.

[99] Willumsen J.F., Simmank K., Filteau S.M., Wagstaff L.A., Tomkins A.M. (1997). Toxic damage to the respiratory epithelium induces acute changes in vitamin A metabolism without depleting retinol stores of South African children. J. Nutr..

[100] Donnen P., Dramaix M., Brasseur D., Bitwe R., Bisimwa G., Hennart P. (2001). The molar ratio of serum retinol-binding protein (RBP) to transthyretin (TTR) is not useful to assess vitamin A status during infection in hospitalized children. Eur. J. Clin. Nut..

[101] Bataille S., Landrier J.F., Astier J., Cado S., Sallette J., Serveaux M., Burtey S., Cohen J., Tournier C., Tournaire F. (2017). Plasma retinol concentration is mainly driven by transthyretin in hemodialysis patients. J. Ren. Nutr..

[102] Vaz-Rodrigues R., Mazuecos L., Villar M., Urra J.M., Gortázar C., de la Fuente J. (2023). Serum biomarkers for nutritional status as predictors in COVID-19 patients before and after vaccination. J. Funct. Foods.

[103] WHO (2008). Worldwide Prevalence of Anaemia 1995–2005. WHO Global Database on Anaemia.

[104] Suchdev P.S., Williams A.M., Mei Z., Flores-Ayala R., Pasricha S.R., Rogers L.M., Namaste S. (2017). Assessment of iron status in settings of inflammation: Challenges and potential approaches. Am. J. Clin. Nutr..

[105] Dagg J.H., Smith J.A., Goldberg A. (1966). Urinary excretion of iron. Clin. Sci..

[106] Howard R.L., Buddington B., Alfrey A.C. (1991). Urinary albumin, transferrin and iron excretion in diabetic patients. Kidney Int..

[107] Bernard A., Ouled Amor A.A., Goemaere-Vanneste J., Antoine J.L., Lauwerijs R.R., Lambert A., Vandeleene B. (1988). Microtransferrinuria is a more sensitive indicator of early glomerular damage in diabetes than microalbuminuria. Clin. Chem..

[108] MacGillivray R.T., Mendez E., Sinha S.K., Sutton M.R., Lineback-Zins J., Brew K. (1982). The complete amino acid sequence of human serum transferrin. Proc. Natl. Acad. Sci. USA.

[109] Donovan A., Lima C.A., Pinkus J.L., Pinkus G.S., Zon L.I., Robine S., Andrews N.C. (2005). The iron exporter *ferroportin/Slc40a1* is essential for iron homeostasis. Cell Metab..

[110] Nemeth E., Tuttle M.S., Powelson J., Vaughn M.B., Donovan A., McVey Ward D., Ganz T., Kaplan J. (2004). Hepcidin regulates cellular iron efflux by binding to ferroportin and inducing its internalization. Science.

[111] Ganz T. (2003). Hepcidin, a key regulator of iron metabolism and mediator of anemia of inflammation. Blood.

[112] Weiss G., Goodnough L.T. (2005). Anemia of chronic disease. N. Engl. J. Med..

[113] Papanikolaou G., Tzilianos M., Christakis J.I., Bogdanos D., Tsimirika K., MacFarlane J., Goldberg Y.P., Sakellaropoulos N., Ganz T., Nemeth E. (2005). Hepcidin in iron overload disorders. Blood.

[114] Wang W., Knovich M.A., Coffman L.G., Torti F.M., Torti S.V. (2010). Serum ferritin: Past, present and future. Biochim. Biophys. Acta.

[115] Thurnham D.I. (2014). Interactions between nutrition and immune function: Using inflammation biomarkers to interpret micronutrient status. Proc. Nutr. Soc..

[116] Rubin L.P., Ross A.C., Stephensen C.B., Bohn T., Tanumihardjo S.A. (2017). Metabolic effects of inflammation on vitamin A and carotenoids in humans and animal models. Adv. Nutr..

[117] Wagh V.D., Deore B.R. (2015). Ready to use therapeutic food (RUTF): An overview. Adv. Life Sci. Health.

[118] Thurnham D.I., Northrop-Clewes C.A., Knowles J. (2015). The use of adjustment factors to address the impact of inflammation on vitamin A and iron status in humans. J. Nutr..

[119] Larson L.M., Namaste S.M., Williams A.M., Engle-Stone R., Addo O.Y., Suchdev P.S., Wirth J.P., Temple V., Serdula M., Northrop-Clewes C.A. (2017). Adjusting retinol-binding protein concentrations for inflammation: Biomarkers reflecting inflammation and nutritional determinants of anemia (BRINDA) project. Am. J. Clin. Nutr..

[120] Rohner F., Namaste S.M., Larson L.M., Addo O.Y., Mei Z., Suchdev P.S., Williams A.M., Sakr Ashour F.A., Rawat R., Raiten D.J. (2017). Adjusting soluble transferrin receptor concentrations for inflammation: Biomarkers reflecting inflammation and nutritional determinants of anemia(BRINDA)project. Am. J. Clin. Nutr..

[121] Namaste S.M., Rohner F., Huang J., Bhushan N.L., Flores Ayala R., Kupka R., Mei Z., Rawat R., Williams A.M., Raiten D.J. (2017). Adjusting ferritin concentrations for inflammation: Biomarkers reflecting inflammation and nutritional determinants of anemia (BRINDA) project. Am. J. Clin. Nutr..

[122] Lynch S.R., Dassenko S.A., Cook J., Juillerat M.A., Hurrell R.F. (1994). Inhibitory effect of a soybean-protein-related moiety on iron absorption in humans. Am. J. Clin. Nutr..

[123] Schmidt J.A., Rinaldi S., Scalbert A., Ferrari P., Achaintre D., Gunter M.J., Appleby P.N., Key T., Travis R.C. (2016). Plasma concentrations and intakes of amino acids in male meat-eaters, vegetarians and vegans: A cross-sectional analysis in the EPIC-Oxford cohort. Eur. J. Clin. Nutr..

[124] Forbes G.B. (1974). Stature and lean body mass. Am. J. Clin. Nutr..

[125] Dewey K.G., Beaton G., Field C., Lonnerdal B., Reeds P. (1996). Protein requirements of infants and children. Eur. J. Clin. Nutr..

[126] Young V.R., Borgonha S. (2000). Nitrogen and amino acid requirements: The Massachusetts Institute of Technology amino acid requirement pattern. J. Nutr..

[127] Rose W.C., Waterlow J.C., Stephen J.M.L. (1957). Human Protein Requirements and their fulfillment in Practice, Proceedings of a Conference in Princetown, USA, 1955.

[128] Mak T.N., Angeles-Agdeppa I., Tassy M., Capanzana M.V., Offord E.A. (2020). The nutritional impact of milk beverages in reducing nutrient inadequacy among children aged one to five years of age in the Philippines: A dietary modelling study. Nutrients.

[129] Parikh P., Semba R., Manary M., Swaminathan S., Udomkesmalee E., Bos R., Poh B.K., Rojroongwasinkul N., Geurts J., Sekartini R. (2022). Animal source foods, rich in essential amino acids, are important for linear growth and development of young children in low- and middle-income countries. Matern. Child Nutr..

[130] Tadesse S.E., Zerga A.A., Mekonnen T.C., Tadesse A.W., Hussien F.M., Feleke Y.W., Anagaw M., Ayele F.Y. (2022). Burden and determinants of anemia among under-five children in Africa: Systematic review and meta-analysis. Anemia.

[131] Kateera F., Ingabire C.M., Hakizimana E., Kalinda P., Mens P.F., Grobusch M.P., Mutesa L., van Vugt M. (2015). Malaria, anaemia and under-nutrition: Three frequently co-existing conditions among preschool children in rural Rwanda. Malar. J..

[132] Diouf S., Sylla A., Diop F., Diallo A., Sarr M. (2013). Anemia among apparently healthy Senegalese children. Arch. Pediatr..

[133] Gedfie S., Getawa S., Melku M. (2022). Prevalence and associated factors of iron deficiency and iron deficiency anemia among under-5 children: A systematic review and meta-analysis. Glob. Pediatr. Health.

[134] Bahizire E., Bahwere P., Donnen P., Tugirimana P.L., Balol’ebwami S., Dramaix M., Nfundiko C., Chirimwami R., Mubamgwa K. (2017). High prevalence of anemia but low level of iron deficiency in preschool children during a low transmission period of malaria in rural Kivu, Democratic Republic of the Congo. Am. J. Trop. Med. Hyg..

[135] Kangas S.T., Salpéteur C., Nikièma V., Talley L., Briend A., Ritz C., Friis H., Kaestel P. (2020). Vitamin A and iron status of children before and after treament of uncomplicated severe acute malnutrition. Clin. Nutr..

[136] González-Fernández D., Nemeth E., Pons E.d.C., Sinisterra O.T., Rueda D., Starr L., Sangkhae V., Murillo E., Scott M.E., Koski K.G. (2022). Multiple indicators of undernutrition, infection, and inflammation in lactating women are associated with maternal iron status and infant anthropometry in Panama: The MINDI cohort. Nutrients.

[137] Keller U. (2019). Nutritional laboratory markers in malnutrition. J. Clin. Med..

[138] Ingenbleek Y., Van den Schriek H.G., De Nayer P., De Visscher M. (1975). Albumin, transferrin, and the thyroxine-binding prealbumin/retinol-binding protein complex in assessment of malnutrition. Clin. Chim. Acta.

[139] Ingenbleek Y., Young V.R. (1994). Transthyretin (prealbumin) in health and disease: Nutritional implications. Annu. Rev. Nutr..

[140] McLaren D.S. (1974). The Great Protein Fiasco. Lancet.

[141] Semba R.D. (2016). The rise and fall of protein malnutrition in global health. Ann. Nutr. Metab..

[142] Williams C.D. (1933). A nutritional disease of childhood associated with a maize diet. Arch. Dis. Child..

[143] Brock J.F., Autret M. (1952). Kwashiorkor in Africa.

[144] Evans D.C., Corkins M.R., Malone A., Miller S., Mogensen K.M., Guenter P., Jensen G.L., ASPEN Malnutrition Committee (2021). The use of visceral proteins as nutrition markers: An ASPEN position paper. Nutr. Clin. Pract..

[145] Lacy M., Roesch J., Langsjoen J. (2019). Things we do for no reason: Prealbumin testing to diagnose malnutrition in the hospitalized patient. J. Hosp. Med..

[146] Zuo P., Tong S., Yan Q., Cheng L., Li Y., Song K., Chen Y., Dai Y., Gao H., Zhang C. (2020). Decreased prealbumin level is associated with increased risk for mortality in elderly hospitalized patients with COVID-19. Nutrition.

[147] Bae H.J., Lee H.J., Han D.S., Suh Y.S., Lee Y.H., Lee H.S., Cho J.J., Kong S.H., Yang H.K. (2011). Prealbumin levels as a useful marker for predicting infectious complicationhs after gastric surgery. J. Gastrointest. Surg..

[148] Ando Y. (2009). Transthyretin: It’s miracle function and pathogenesis. Rinsho Byon.

